# “Terror Birds” (Phorusrhacidae) from the Eocene of Europe Imply Trans-Tethys Dispersal

**DOI:** 10.1371/journal.pone.0080357

**Published:** 2013-11-27

**Authors:** Delphine Angst, Eric Buffetaut, Christophe Lécuyer, Romain Amiot

**Affiliations:** 1 Laboratoire de Géologie de Lyon : Terre, Planètes, Environnement UMR CNRS/Univ. Lyon 1/ENS-Lyon 5276 Département des Sciences de la Terre Faculté des Sciences, Université Lyon 1 Campus de la Doua, Villeurbanne, France; 2 Centre National de la Recherche Scientifique, UMR 8538, Laboratoire de Géologie de l’Ecole Normale Supérieure, Paris, France; 3 Laboratoire de Géologie de Lyon : Terre, Planètes, Environnement UMR CNRS/Univ. Lyon 1/ENS-Lyon 5276 Département des Sciences de la Terre Faculté des Sciences, Université Lyon 1 Campus de la Doua, Villeurbanne, France, also at Institut Universitaire de France; Raymond M. Alf Museum of Paleontology, United States of America

## Abstract

**Background:**

Phorusrhacidae was a clade including middle-sized to giant terrestrial carnivorous birds, known mainly from the Cenozoic of South America, but also occurring in the Plio-Pleistocene of North America and the Eocene of Africa. Previous reports of small phorusrhacids in the Paleogene of Europe have been dismissed as based on non-phorusrhacid material.

**Methodology:**

we have re-examined specimens of large terrestrial birds from the Eocene (late Lutetian) of France and Switzerland previously referred to gastornithids and ratites and have identified them as belonging to a phorusrhacid for which the name *Eleutherornis cotei* should be used.

**Conclusions/Significance:**

The occurrence of a phorusrhacid in the late Lutetian of Europe indicates that these flightless birds had a wider geographical distribution than previously recognized. The likeliest interpretation is that they dispersed from Africa, where the group is known in the Eocene, which implies crossing the Tethys Sea. The Early Tertiary distribution of phorusrhacids can be best explained by transoceanic dispersal, across both the South Atlantic and the Tethys.

## Introduction

The Phorusrhacidae (the so-called “terror birds”) were terrestrial carnivorous birds, ranging in height from about 90 cm to more than 2 m, which are a highly distinctive element of the Cenozoic faunas of South America [1]–[4].They reached North America in the Pliocene during the Great American Biotic Interchange following the formation of the Isthmus of Panama [5]. Small phorusrhacids were reported from the Palaeogene of Europe [6], [7], but these records were actually based on non-phorusrhacid material [1], [8], [9]. Similarly, reports from the Palaeogene of North America [10] have been dismissed [1]. The only well supported record of a phorusrhacid from the Old World hitherto was a femur from the late Early or early Middle Eocene of Algeria described as *Lavocatavis africana* [11]. The re-examination of avian fossils from two Middle Eocene localities, in France and Switzerland, previously referred to other groups of giant birds, shows that phorusrhacids were indeed present in Europe during the Palaeogene. Their presence there at this early date raises interesting questions about the geographical origin and subsequent biogeographical history of the Phorusrhacidae and about their possible ecological interactions with other extinct giant birds.

## Results

### Systematic Palaeontology

Aves Linnaeus, 1758

Cariamae Fürbringer, 1888

Phorusrhacidae Ameghino, 1889

Psilopterinae Dolgopol de Saez, 1927

Genus *Eleutherornis* Schaub, 1940

*Eleutherornis cotei* (Gaillard, 1936), new combination

Diagnosis: A middle-sized phorusrhacid (height about 1.5 m) showing a combination of basal and derived characters. Trochlea II of the tarsometatarsus is expanded medially as in psilopterines, while the pre-acetabular part of the ilia is more compressed laterally and more closely appressed to the neural spines of the synsacral vertebrae than is usual in psilopterines and more reminiscent of more derived phorusrhacids.

**Figure 1:**
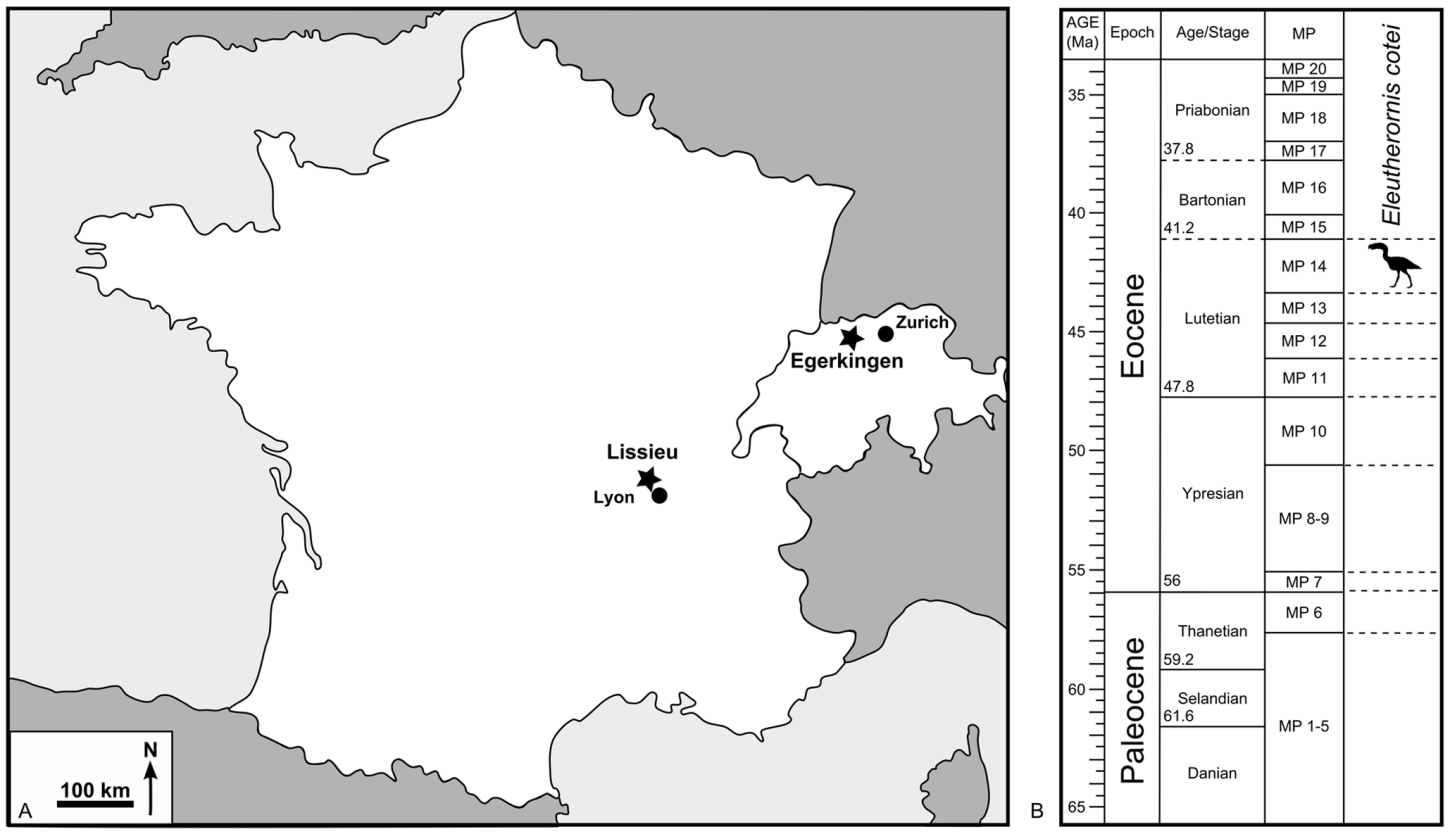
Geographical and stratigraphic setting of *Eleutherornis cotei*: A) Geographical location of the sites of Lissieu (France) and Egerkingen (Switzerland); B) Stratigraphic level of *Eleutherornis cotei* from Lissieu and Egerkingen.

Type locality: Fissure filling at Lissieu (Rhône, France) (Fig. 1).

Stratigraphic provenance: Middle Eocene, late Lutetian, MP14 mammalian reference level (approximately 43.5 to 41.2 ma [12]) (Fig. 1).

Type material: Distal end of tarsometatarsus (here designated as the lectotype) (L71). Hypodigm: trochlea III of a tarsometatarsus (L72), phalanges (L66, L67), ungual phalanges (L68, L69, L70, L73).

Supplementary material from Lissieu: Trochlea III of a tarsometatarsus (FSL 337282, L467, L474), ungual phalanges (FSL 337281, L135, L468, L469, L470, L471, L472, L473).

Referred material from Egerkingen (canton Solothurn, Switzerland): anterior part of a pelvis (synsacrum and ilia) (Eh.781); posterior part of a synsacrum (Eh. 782); phalanges (Ef. 998, Ef. 999, Ef. 1000), ungual phalanges (Ef. 1001 and unnumbered).

No permits were required for the described study, which complied with all relevant regulations.

The specimens described in the present study are part of three public paleontological collections (Musée des Confluences, Lyon, France; Naturhistorisches Museum Basel, Switzerland; Université Claude Bernard-Lyon 1, France) and were collected decades ago. Permission to study them was granted by the curators in charge (as mentioned in the acknowledgments) and all collection numbers are provided in the text. No field work was involved in the present study, therefore no permits were required.

### Comparative description and identification

**Figure 2:**
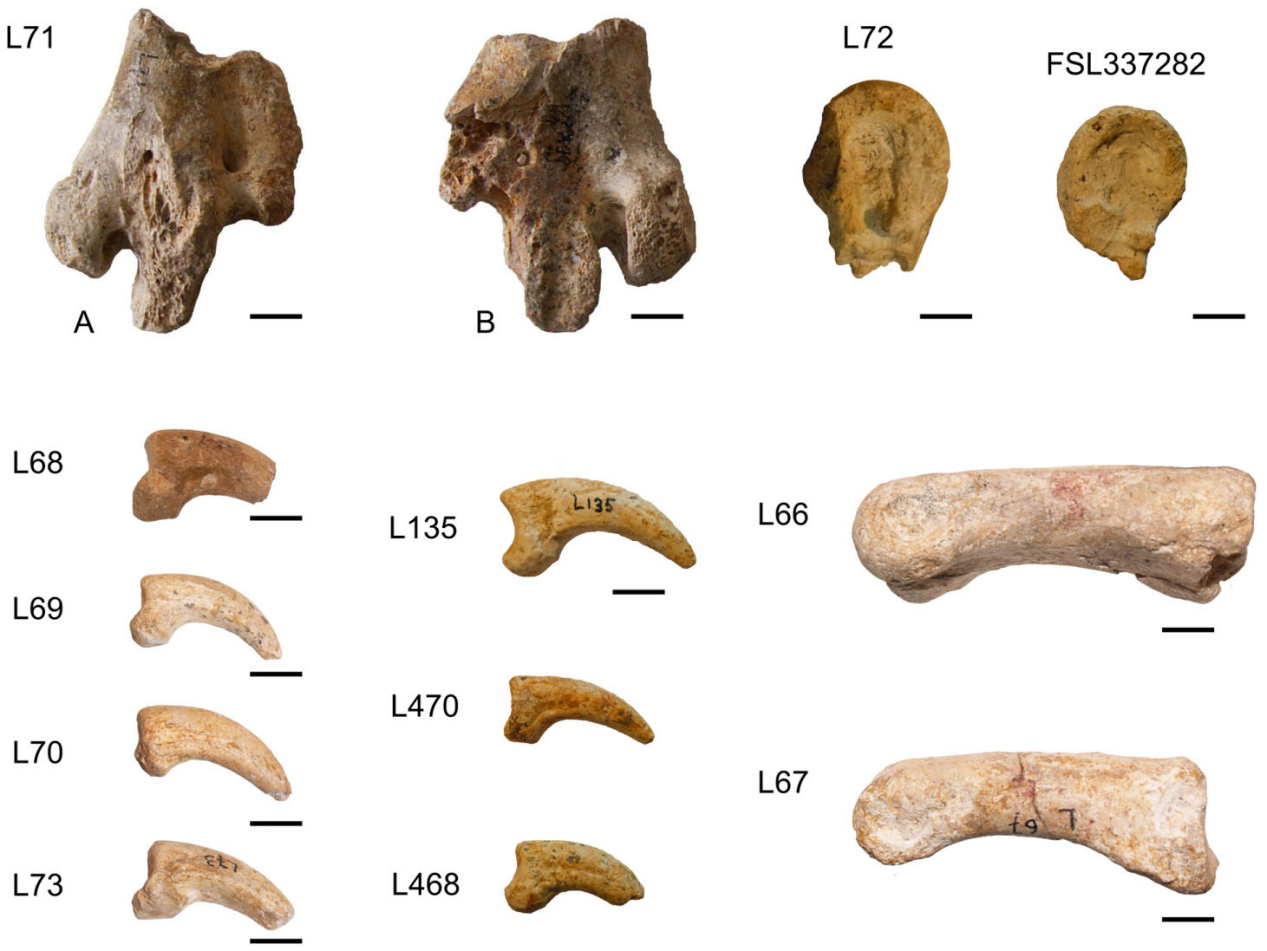
*Eleutherornis cotei* material from Lissieu. L71: distal end of left tarsometatarsus (lectotype), A) cranial view, B) caudal view; L72, FSL337282: left median (III) trochleae in lateral view; L68, L69, L70, L73, L135, L470, L468: ungual phalanges; L66, L67: phalanges. L66, L67, L68, L69, L70, L71, L72, and L73: type series described by Gaillard (1937) kept in the Musée des Confluences, Lyon. FSL337282: supplementary material kept in the University Lyon 1, Lyon L135, L470, and L468: supplementary material kept in the Musée des Confluences, Lyon. All scale bars are 1 cm.

**Figure 3:**
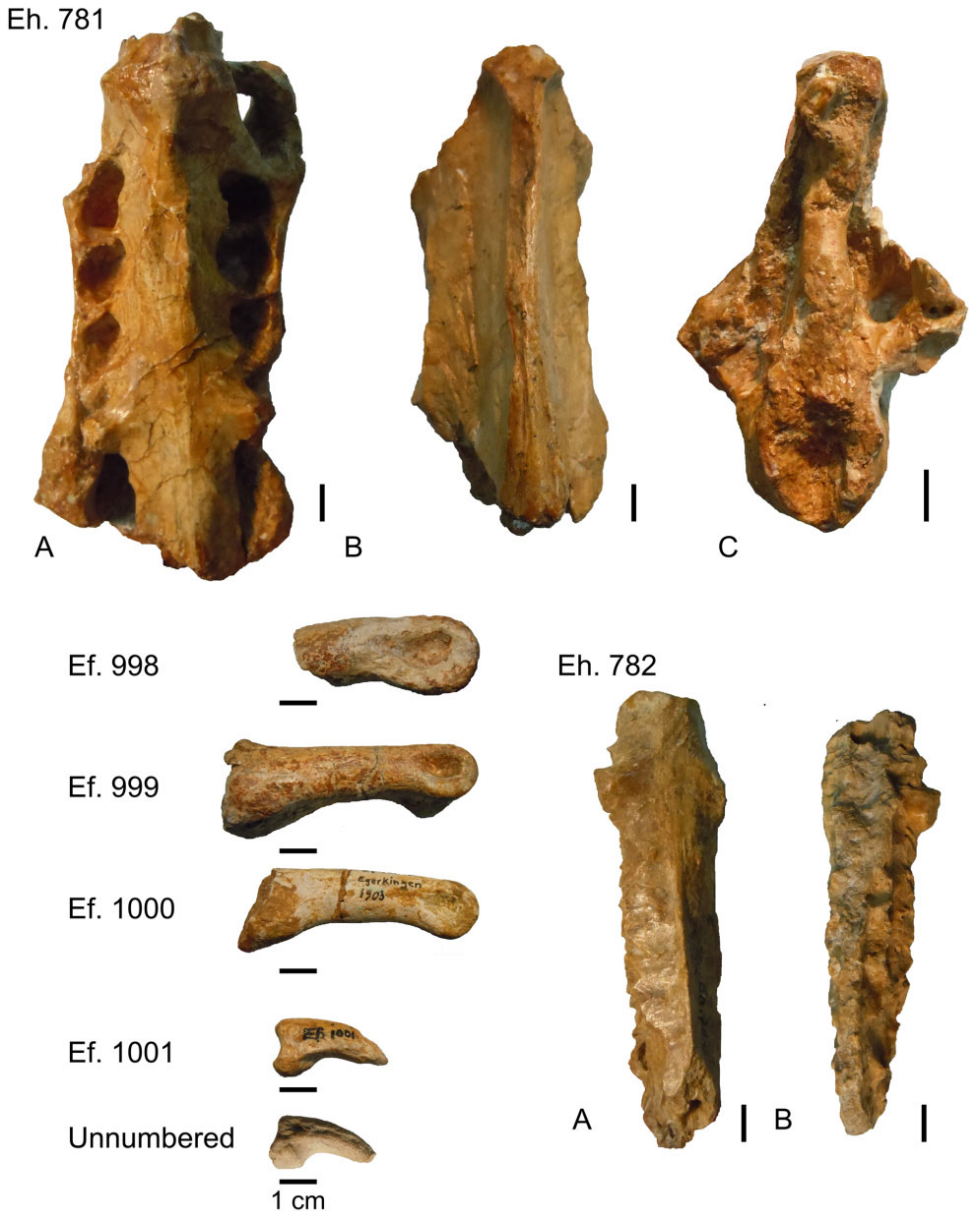
*Eleutherornis cotei* material from Egerkingen. Eh.781: anterior part of a pelvis (synsacrum and ilia), A) ventral view, B) dorsal view, C) cranial view; Eh.782: posterior part of a synsacrum, A) ventral view, B) dorsal view. Ef. 998, Ef. 999, Ef. 1000: phalanges; Ef. 1001 and unnumbered: ungual phalanges. Scale bars : 1 cm.

**Figure 4:**
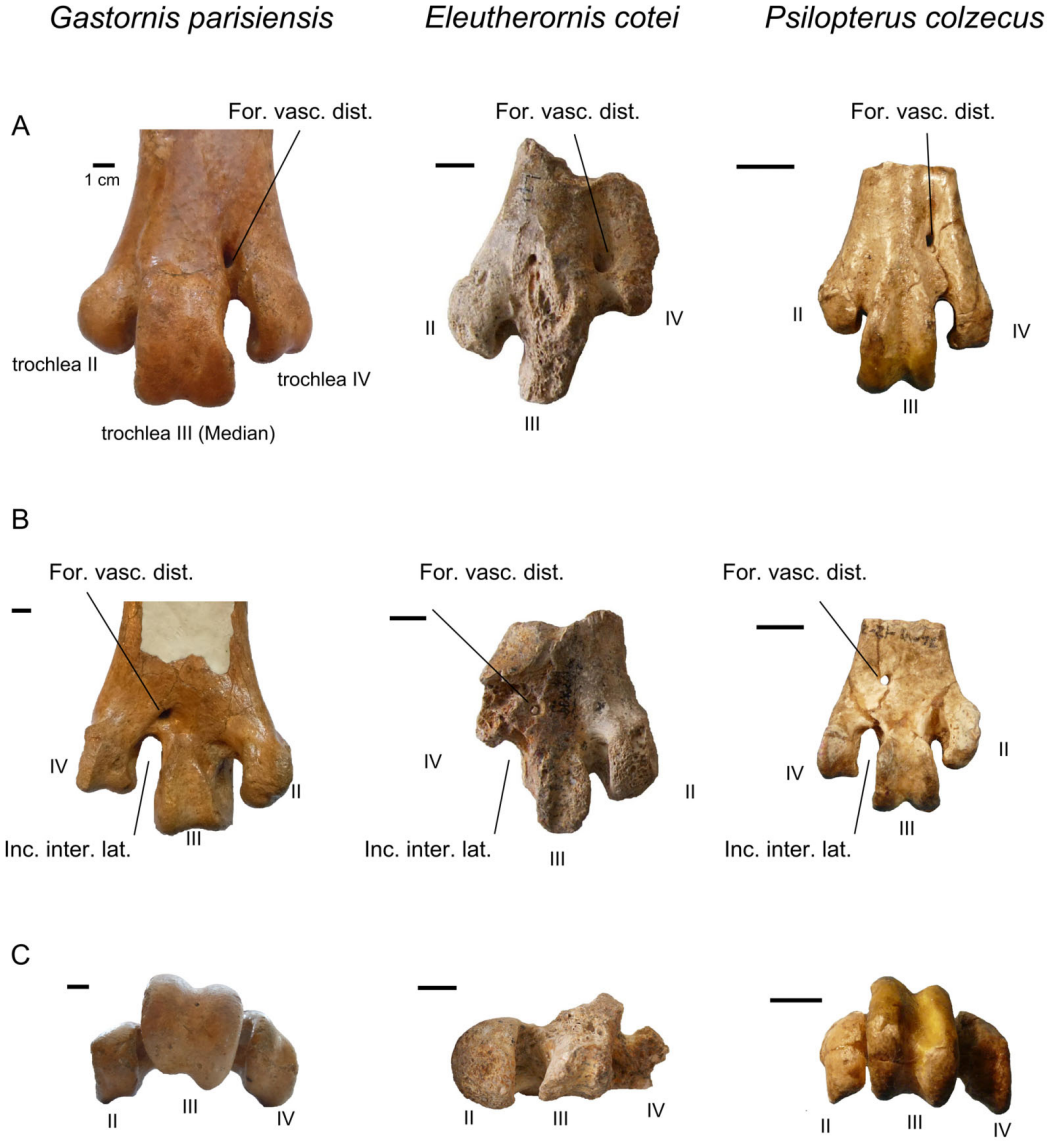
Comparison of the distal end of the tarsometatarsus in *Gastornis parisiensis, Eleutherornis cotei* and *Psilopterus colzecus*. A) cranial view, B) caudal view, C) distal view. For. Vasc. Dist.: foramen vasculare distale, Inc. Inter. Lat.: Incisura intertrochlearis lateralis. Scale bars: 1 cm.

**Figure 5:**
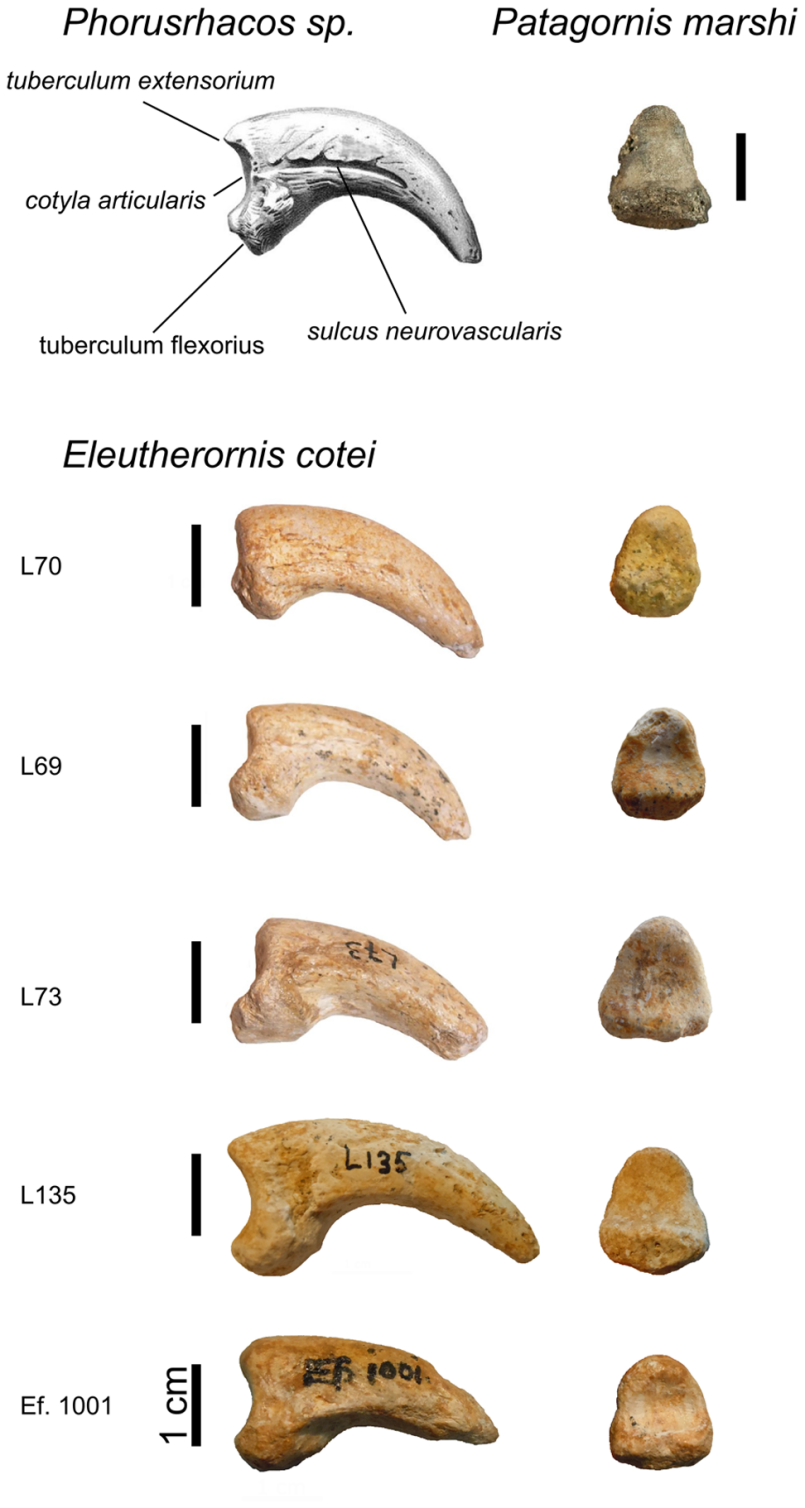
Comparisons of the ungual phalanges of South American phorusrhacids (*Phorusrhacos* sp. and *Patagornis marshi*) and *Eleutherornis cotei*. Scale bars: 1 cm.

The material from Lissieu was described in detail [13], [14] as *Diatryma*? *cotei* (Fig. 2). It was later considered as a genus *incertae sedis* [17]. From Egerkingen, phalanges were described first, as Avis *incertae sedis* [15]; the taxon *Eleutherornis helveticus* was later erected on the basis of pelvic remains (Fig. 3), which were considered as belonging to a ratite [16]. Possible phorusrhacid affinities have been suggested but not supported [17]. The specimens from Lissieu show no real similarities with “*Diatryma*” (a junior synonym of *Gastornis* [18]) or with gastornithids in general, as already noted by Andors [19]. Besides being considerably smaller than *Gastornis* specimens, the tarsometatarsus from Lissieu clearly differs from that genus in its foramen vasculare distale, which bifurcates into two channels completely enclosed in bone, one of which opens on the caudal surface of the bone, while the other one opens at the bottom of the incisura intertrochlearis lateralis (Fig. 4). In gastornithids, a single bone-enclosed channel issues from the foramen, opening on the caudal face of the bone, and an open groove connects the foramen to the incisura. The claw-like, hooked ungual phalanges from Lissieu, with a strong proximally directed flexor tubercle, are unlike the rather hoof-shaped ungual phalanges of *Gastornis* [19] (Fig. 5).

**Figure 6:**
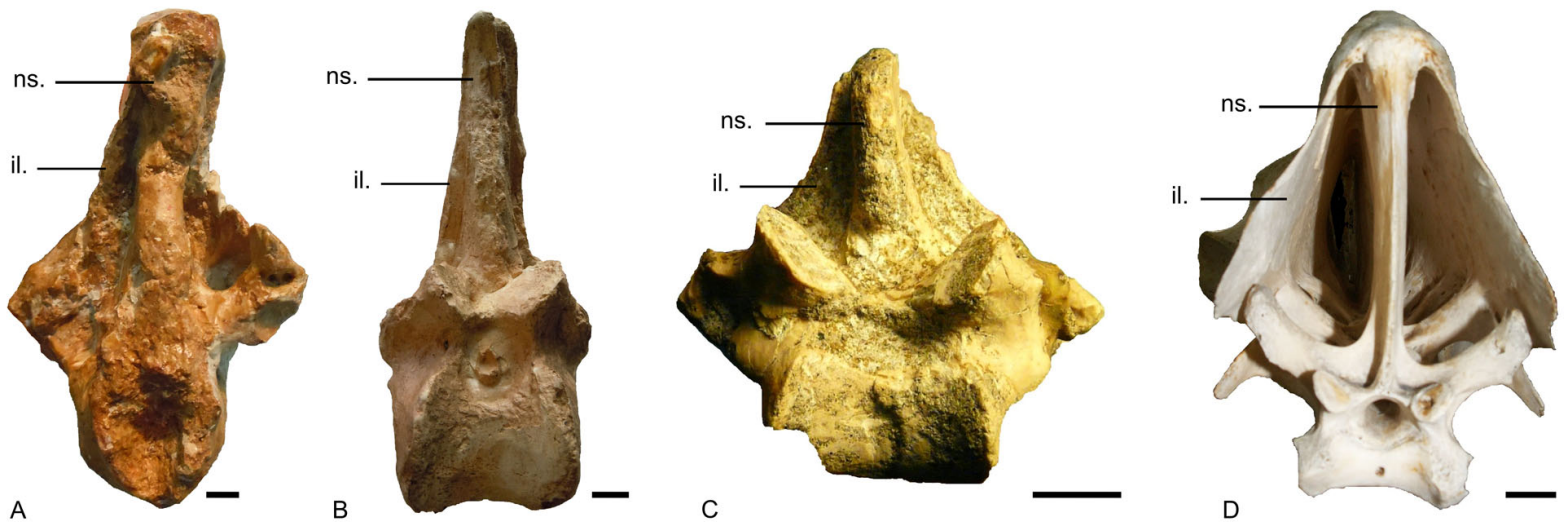
Comparison of the pelves (in cranial view) of *Eleutherornis cotei* (A), *Andalgalornis steulleti* (B), *Procariama simplex* (C) and *Struthio camelus* (D). ns.: neural spine, il.: ilium. Photograph of *Procariama simplex* courtesy of Federico Agnolin. Scale bars: 1 cm.

The main pelvic fragment from Egerkingen, consisting of the cranial part of the synsacrum and the ilia, differs from that of ratites in its stronger lateral compression and the fact that the ilia are closely appressed to the neural spines of the anterior synsacral vertebrae (Fig. 6). In ratites, the ilia contacts the neural spines at their top but strongly diverge ventrally, forming in cranial view a tent-like structure that is unlike the much narrower condition seen in the Egerkingen fragment.

Phorusrhacid characters in the material from Lissieu and Egerkingen include:

  - The condition of the foramen vasculare distale (see above) and the general morphology of the trochleae on the tarsometatarsus from Lissieu (Fig. 4).
  - The morphology of the ungual phalanges from both Lissieu and Egerkingen. They are strongly recurved and hook-shaped, with grooves along the sides and a well-developed flexor tubercle. As in phorusrhacids, the tubercle is oriented proximally, unlike the condition in raptors, where it is oriented distally. The proximal articular face in the phalanges from Lissieu and Egerkingen widens ventrally as in phorusrhacids (Fig. 5) [20]. The ungual phalanges from Lissieu and Egerkingen exhibit all the characters listed by Cenizo et al.[21] for phorusrhacid.
  - The strong lateral compression of the anterior part of the pelvis from Egerkingen. As mentioned above, the ilia are closely appressed to the neural spines of the synsacral vertebrae, which is a phorusrhacid character [3]. The general shape of the ilia, forming a narrow anterior ridge which begins to broaden at the level of the acetabulum, is also phorusrhacid-like (Fig. 6).

Because of the above-mentioned close similarities of the various skeletal elements from Lissieu and Egerkingen, they are referred here to phorusrhacids. Comparison with other Eocene birds from Europe, notably those erroneously referred to phorusrhacids such as *Strigogyps* [8], do not reveal significant similarities. In particular, the ungual phalanges of the significantly smaller *Strigogyps* are more compressed laterally and have a less developed flexor tubercle. The only elements among the French and Swiss material that can be compared directly are the ungual phalanges and they are virtually identical. Other elements are compatible in size, indicating a bird roughly the size of the phorusrhacid *Patagornis marshi*, from the Miocene of Patagonia (height about 1.5 m). In view of the fact that the specimens come from two localities of the same geological age (late Lutetian), which are not very distant geographically (about 300 km) (Fig. 1) and were part of the same land mass in the Eocene, the most parsimonious interpretation is to refer all of the material to a single taxon, using Schaub’s genus name and Gaillard’s species name, namely *Eleutherornis cotei* (Gaillard, 1936). It had already been suggested [9] that the bird from Lissieu and that from Egerkingen could belong to the same clade, but they were then placed among the palaeognaths.

Although it is known from fairly incomplete material, *Eleutherornis cotei* can be distinguished from other phorusrhacids by a combination of characters. The tarsometatarsus is reminiscent of the Psilopterinae, a subfamily of mostly relatively small and basal phorusrhacids, by the shape of trochlea II, which is relatively broad and rounded [22] (medial expansion of trochlea II is considered as a diagnostic character of Psilopterinae [3]), unlike the condition in more derived phorusrhacids such as *Patagornis* [23] or *Phorusrhacos* [22], in which trochlea II is narrower and more parallel to trochlea III. In addition, in distal view the trochleae are more or less in the same plane, which is plesiomorphic for phorusrhacids [2]. It cannot be determined whether in *Eleutherornis cotei* trochlea II bore a caudal process, as in psilopterines, because that area is broken and worn. *Eleutherornis cotei*, however, is significantly larger than other psilopterines, being about the size of *Patagornis marshi.* The cranial part of the pelvis appears to be more compressed laterally than in most psilopterines, notably *Procariama*, in which the ilia are more divergent and less closely appressed to the neural spines. Although it appears to be relatively basal in some respects, *Eleutherornis cotei* also shows some derived features (including a relatively large size), resulting in a combination of characters not known in any other representative of the group. It is provisionally placed in the subfamily Psilopterinae mainly because of the characters of the tarsometatarsus.

### The phorusrhacid record in Europe

The remains of *Eleutherornis cotei* described in this study come from two sites in Western Europe that are similar in various ways. The French specimens are from a fissure filling at Lissieu (Rhône), a few kilometres north of the city of Lyon (Fig.1). The locality, which was found in the late 19^th^ century [13], [14], [24]–[26] and no longer exists because of quarry exploitation, yielded a diverse vertebrate fauna. The mammal assemblage corresponds to the MP14 mammalian reference level [27], considered as equivalent to the late Lutetian [12].

The Swiss material comes from the fissure fillings at Egerkingen (canton Solothurn), which have been known since the 19^th^ century for their abundant mammal remains [28] (Fig. 1). *Eleutherornis* remains come from two of the Egerkingen fissures, γ and α, which may not be exactly of the same age [27], but are not far distant in time (J. Hooker, pers. com.) and correspond to MP14.

All the remains referred to *Eleutherornis cotei* are thus from similar geological settings (fissure fillings, perhaps suggesting an upland habitat) and of the same age, viz. late Lutetian (MP14). It is worth noting that the specimens from Lissieu and Egerkingen are currently the only well-attested records of phorusrhacids from Europe. The Palaeogene avian fossil record of Europe is of good quality [9], [17], [29] and there seems to be no evidence of phorusrhacids either before or after MP14. Older localities yielding fossil birds in some abundance, such as Messel and the Geiseltal, have not yielded phorusrhacid remains, and they are not known from the younger karstic sites of the Quercy, in which bird remains are common. This may suggest a brief incursion of this group of giant birds on the European continent at the end of the middle Eocene, followed by local extinction. The main group of large terrestrial birds in Europe from the middle Palaeocene to the middle Eocene was the family Gastornithidae, but the latest known representatives of the group appear to be from the obere Mittelkohle of the Geiseltal (Germany), which corresponds to MP13 [30]. Therefore, there is no evidence that phorusrhacids and gastornithids coexisted or competed in Europe.

### Palaeobiogeographical implications

For a long time phorusrhacid birds were considered as an essentially South American group, the late Cenozoic North American forms being easily explained by dispersal from South America via the isthmus of Panama during the Great American Biotic Interchange. However, because of the recent discovery of a phorusrhacid in the Eocene of Africa and the description of phorusrhacids from the Middle Eocene of Europe in the present paper, a reappraisal of phorusrhacid biogeography is needed. The topic was already discussed in the 1980s when purported phorusrhacids were reported from the Palaeogene of Europe, but the subject was dropped when it became apparent that the fossils in question belong to other taxonomic groups. It is now possible to reconsider the question on the basis of solid evidence for the presence of phorusrhacids in Europe during a well-defined period of the Eocene, and of the occurrence of the group in the Eocene of Africa.

**Figure 7:**
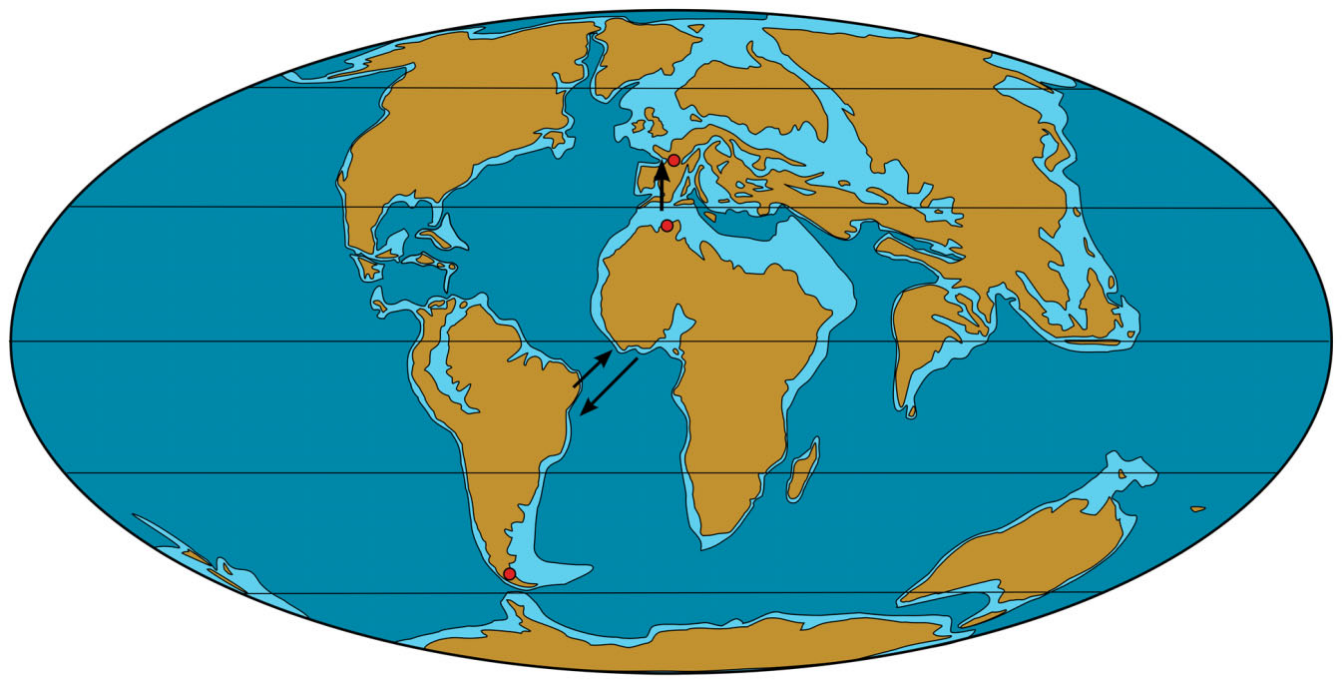
Paleobiogeographical distribution of the Phorusrhacidae in the Eocene. Arrows: likely trans-oceanic dispersal routes of the Phorusrhacidae.

The place of origin of the Phorusrhacidae is a moot point. As already pointed out [4], their greatest diversification was in South America, but that does not exclude a possible origin outside that continent. The earliest record of a phorusrhacid is *Paleopsilopterus itaboraiensis*, from the Palaeocene of Brazil [1], [3], [31]. However, doubt has been cast about the real systematic position of this taxon [2], [4], [32], and its palaeobiogeographical significance is therefore doubtful. The earliest undoubted phorusrhacids are from the Eocene, as follows (Fig. 7):

  - An unnamed psilopterine from the Vacan (probably early Eocene) of Cañadon Vaca, in Patagonia [4], [33].
  - *Lavocatavis africana*, from the late Early or early Middle Eocene of Algeria [11].
  - *Eleutherornis cotei*, from the Middle Eocene (late Lutetian, MP14) of France and Switzerland.

In terms of geological age, these early records of phorusrhacids are thus not very distant from one another and it would be unsafe to draw conclusions about the place of origin of the group simply from its possible earliest occurrence. However, as mentioned above, the European record does not suggest that phorusrhacids were present there before MP14, so that a European origin is unlikely. The situation in Africa is more difficult to assess, because the Palaeogene record of terrestrial birds on that continent is very scanty, and it is not possible at the moment to estimate how long phorusrhacids may have been present on that continent.

If, as suggested by their stratigraphic distribution, European phorusrhacids are considered as immigrants from elsewhere rather than having originated on that continent, a first point to be considered is how dispersal may have taken place. The question of the possible flight abilities of some phorusrhacids is important from that point of view. There is no doubt that the larger forms of phorusrhacids, with reached a very large size and had strongly reduced wings, were flightless. Things are less clear for the smaller psilopterines. It has been suggested that psilopterines were able to fly [33], [34], but this has been contested [1], [3]. In any case, the relatively large *Lavocatavis* was in all likelihood flightless [11] and so was the similarly sized *Eleutherornis*. Therefore, flight across marine barriers probably cannot explain the dispersal of the African and European phorusrhacids, unless one assumes that they developed flightlessness independently from flying ancestors, a hypothesis that has already been discussed in the case of *Lavocatavis* and considered unlikely because of complete morphological similarity between the African form and those from South America [4]. This also applies to *Eleutherornis*. Parallel or convergent evolution can thus be excluded.

Several possibilities can be suggested for the origin and dispersal of phorusrhacids (Fig. 7):

  - Direct dispersal from South America to Europe is unlikely because of distance and lack of likely direct terrestrial connections across the central Atlantic.
  - Dispersal from South America to Europe via North America and a North Atlantic connection has been considered from some groups of terrestrial vertebrates, including phorusrhacids [35]. It seems unlikely in the case of phorusrhacids because purported representatives of the group from the Eocene of North America have been dismissed [1] and the rather good Palaeogene avian fossil record from that continent shows no trace of them.
  - The likeliest origin for the European phorusrhacids is therefore Africa. Dispersal from Africa to Europe in the Eocene involved crossing the Tethys Sea. Several dispersal phases from Africa to Laurasia during the Palaeogene, involving land vertebrates, have been identified or postulated [36], including a doubtful one at the Ypresian-Lutetian boundary, which is probably too early to account for the dispersal of phorusrhacids, and an equally doubtful one towards the Lutetian-Bartonian boundary, which may better fit their known stratigraphic distribution in Europe. Possible dispersal routes across the Tethys may have been provided by the Alboran and Apulian platforms, especially during episodes of low sea levels, with one occurring in the late Lutetian [12].

Irrespective of their dispersal from Africa to Europe, whether phorusrhacids originated in Africa or in South America remains uncertain. As noted above, stratigraphic evidence is inconclusive in this respect. The fact that the greatest diversification of phorusrhacids took place in South America has been considered as suggesting a South American origin [4], [11] but cannot completely rule out an African origin. The presence in South America of the closest relatives of the Phorusrhacidae, the Cariamidae [11] possibly carries more weight. A vicariance model for the evolution of South American and African phorusrhacids is untenable, because it would push back the origin of the group to the Early Cretaceous, before the opening of the South Atlantic Ocean, well before the appearance of the modern groups of birds. Therefore, dispersal across the South Atlantic remains the only likely possibility, whatever the direction in which it took place. A transatlantic crossing by flightless birds is not as unlikely at it may seem, for several reasons:

  - In the early Tertiary, the South Atlantic was significantly narrower than it is today and large islands subsisted along the now-submerged Walvis Ridge and Rio Grande Rise until the Eocene [37]. They provided possible “stepping stones” for island-hopping dispersal.
  - Transatlantic dispersal, either by island-hopping or drafting on “floating islands” is considered likely for a number of vertebrate groups that occur in both Africa and South America, including burrowing amphisbaenian reptiles [38] and various mammals, including rodents [39] and primates [40]. It should be noted, however, that most instances of transatlantic crossing are supposed to have taken place from Africa to South America (perhaps facilitated by sea currents), which may suggest that the Phorusrhacidae originated in Africa and then dispersed to both Europe and South America. The alternative hypothesis is that phorusrhacids firstly appeared in South America and then dispersed to Africa, from where they eventually reached Europe. A detailed analysis of transatlantic dispersal was made by Ezcurra and Agnolin [41]who discussed in detail several clades of shared African-South American organisms.Whatever hypothesis is correct, transatlantic dispersal must have taken place early in the Palaeogene, no later than the Early Eocene.

## Discussion

A reappraisal of various fossil avian remains from France and Switzerland leads to the conclusion that a quite large flightless bird of the family Phorusrhacidae was present in Europe at the end of the Middle Eocene, apparently after the extinction of another family of large terrestrial birds previously well represented in Europe, the Gastornithidae. The European phorusrhacids were in all likelihood derived from African forms, through dispersal across the Tethys Sea. Current stratigraphic evidence suggests that the presence of phorusrhacids was of short duration, apparently being restricted to the late Lutetian (MP14). During this short time span (perhaps not more than 3 million years [12]) phorusrhacids presumably were among the top predators in Europe. Why they failed to diversify and prosper in Europe, unlike what happened in South America where a considerable phorusrhacid radiation took place during the Cenozoic, is not completely clear, but the competition of placental carnivores may have played an important part [9]. It has often been noted [42], [43] that in geographically isolated South America the phorusrhacids radiated in environments where the only other terrestrial predators were terrestrial crocodilians and marsupials, with which they competed successfully. Their demise has sometimes been linked to the massive arrival of Holarctic placental carnivores in South America at the time of the Great American Biotic Interchange [33], [42], although the fact that phorusrhacids dispersed to North America suggests that this may be an oversimplification. Faced in Europe with the competition of other types of predators, phorusrhacids may have been unable to survive. However that may be, the identification of phorusrhacids in the Eocene of France and Switzerland raises interesting questions about the trophic role these large terrestrial carnivorous birds may have played in the ecosystems of the European continent, where predatory mammals (Creodonta, Carnivora) were already well established.
